# Advancements and trends in digestive system autotransplantation: a bibliometric and visualization analysis

**DOI:** 10.3389/fmed.2025.1537446

**Published:** 2025-07-17

**Authors:** Aimitaji Abulaiti, Talaiti Tuergan, Alimu Tulahong, Ruiqing Zhang, Yingmei Shao, Tuerganaili Aji

**Affiliations:** ^1^Hepatobiliary and Echinococcosis Surgery Department, Digestive and Vascular Surgery Center, First Affiliated Hospital of Xinjiang Medical University, Urumqi, China; ^2^State Key Laboratory of Pathogenesis, Prevention and Management of High Incidence Diseases in Central Asia, Xinjiang Medical University, Urumqi, China; ^3^Xinjiang Clinical Research Center for Echinococcosis and Hepatobiliary Diseases, First Affiliated Hospital of Xinjiang Medical University, Urumqi, China

**Keywords:** digestive system autotransplantation, autologous transplantation, pancreatic transplantation, chronic pancreatitis, bibliometric analysis

## Abstract

**Background:**

Digestive system autotransplantation is an emerging surgical technique used to treat complex digestive diseases.

**Methods:**

This study conducted a bibliometric analysis of 748 publications from the Web of Science Core Collection (WoSCC) database, using VOSviewer and CiteSpace tools to map research trends, author contributions, and institutional collaborations. Articles were selected based on their relevance to digestive system autotransplantation, focusing on autologous liver, pancreatic, and small intestine transplantation. The analysis included publication volume, citation counts, key authors, leading journals, and keyword co-occurrence.

**Results:**

The analysis revealed a steady rise in publications between 2004 and 2015, followed by a gradual decline after 2016. The United States leads in research output, accounting for 40.11% of publications, followed by China. The leading institutions are predominantly based in the United States, with the University of Minnesota System producing the most publications. High-frequency keywords include autologous transplantation, pancreatic transplantation, chronic pancreatitis (CP), and postoperative complications.

**Conclusion:**

Digestive system autotransplantation is a promising approach for complex cases. Continued interdisciplinary collaboration and focus on clinical outcomes will drive future advancements.

## Introduction

Digestive system autotransplantation has emerged as an advanced surgical technique, demonstrating significant potential in treating complex digestive system diseases in recent years. This technique includes autologous liver transplantation, pancreatic transplantation, and small intestine transplantation (1–3). Traditional digestive surgeries, such as liver resection, pancreatic resection, and small intestine resection, have achieved some success in treating tumors and other diseases. However, for tumors located in complex anatomical areas adjacent to critical blood vessels, the surgical risks and difficulties are extremely high, often rendering them “unresectable” (4, 5). These unresectable tumors, due to their special locations, make it challenging to completely remove the tumor without damaging vital structures during surgery, thus increasing the risk of postoperative recurrence and complications (6). Advances in surgical techniques, such as vascular occlusion, vena cava shunting, and *ex vivo* resection, have made autotransplantation feasible (7). This technique combines traditional resection and organ transplantation, involving the removal, repair, and reconstruction of the entire organ *ex vivo* in cold preservation, and then reimplanting the unaffected part of the organ (8–10). The development of this technique not only provides new treatment options but also offers surgeons more choices when dealing with complex cases.

Autologous liver transplantation combines the advantages of traditional liver resection and liver transplantation. It provides a new treatment strategy, especially for complex conditions such as multiple liver tumors or hepatic echinococcosis (2, 11). Compared to whole liver transplantation, autotransplantation does not require a donor liver, avoiding the risks of donor shortage and long-term postoperative immunosuppression, while also reducing the risk of disease progression and waiting time for a donor (12). This technique allows for more precise removal of lesions, reducing the risk of uncontrollable bleeding during surgery, increasing the surgical space for complex vascular reconstruction, and minimizing ischemic damage. Despite these advantages, digestive system autotransplantation is highly challenging, requiring a highly skilled surgical team, and has not yet been widely standardized in clinical practice (13, 14). This partly explains why this technique has not been widely adopted. Successful implementation of autotransplantation requires a surgical team with high professional skills and extensive experience, along with precise preoperative assessments and intraoperative support. However, with the development of preoperative three-dimensional image reconstruction technology and liver indocyanine green clearance technology, preoperative estimation of the remnant liver volume has become possible (15–17). This pre-assessment technology, through precise measurement and simulation of intraoperative conditions, greatly improves the safety and success rate of the surgery (11, 18, 19). Hepatic alveolar echinococcosis (HAE) is a severe parasitic infection often leading to extensive liver tissue destruction. Traditional surgery struggles to completely remove the diseased tissue, while autotransplantation provides a new possibility for radical treatment (11). Furthermore, in treating hepatobiliary malignancies, autotransplantation also shows significant advantages by completely removing lesions and preserving as much healthy tissue as possible, significantly improving patient prognosis (20).

Autologous pancreatic transplantation shows unique advantages in treating chronic pancreatitis (CP) and preventing postoperative diabetes (21). Chronic pancreatitis and pancreatic tumors pose significant therapeutic challenges due to their complex anatomical structures and the essential endocrine function of the pancreas (22). Traditional surgical interventions, such as pancreaticoduodenectomy or distal pancreatectomy, can relieve symptoms and remove tumors but often result in substantial loss of pancreatic function, leading to diabetes (23). Autologous pancreatic transplantation, particularly autologous islet transplantation, offers a promising alternative. This technique involves isolating islet cells from the resected pancreas and then autotransplanting them into the liver or other suitable locations in the patient's body. This method helps preserve endocrine function and reduce the risk of postoperative diabetes (21). Autologous islet transplantation is particularly beneficial for patients undergoing total pancreatectomy (TP) for chronic pancreatitis, as it can significantly improve their quality of life by maintaining insulin independence or reducing insulin demand (24, 25). The technical complexity of autologous pancreatic transplantation lies in the isolation and transplantation of islet cells. Advances in islet isolation techniques and immunosuppressive regimens have improved the outcomes of this surgery, making it a viable option for a broader range of patients (25). Preoperative planning, including imaging studies and functional assessments, is crucial for optimizing results and reducing complications.

Autologous small intestine transplantation is primarily used to treat extensive small intestine diseases, such as short bowel syndrome (SBS) and certain complex intestinal tumors (26, 27). The small intestine is crucial for nutrient absorption, and its extensive resection can lead to short bowel syndrome (SBS), characterized by malnutrition, diarrhea, and severe nutritional deficiencies (28). Traditional surgical treatments for SBS are usually limited and may not restore sufficient intestinal function (29). Autologous small intestine transplantation has emerged as a new approach to address these challenges. This surgery involves the resection of the affected small intestine, *ex vivo* treatment, and reimplantation into the patient's body. By preserving and reusing the patient's own small intestine, this technique aims to restore the continuity and function of the intestine, thereby improving the patient's nutritional status and quality of life. Advances in surgical techniques and perioperative care have enhanced the feasibility and effectiveness of autologous small intestine transplantation. Preoperative evaluation, including detailed imaging studies and functional tests, is crucial for ensuring surgical success. Additionally, postoperative care, including nutritional support and monitoring of complications, plays a key role in the long-term success of this approach (30, 31).

In summary, digestive system autotransplantation, as a cutting-edge surgical technique, is gradually becoming an important means of addressing complex digestive system diseases. Through continuous technological innovation and accumulation of clinical practice, this technique is expected to be more widely applied in the future, bringing hope for recovery to more patients. With the strengthening of global medical exchanges and cooperation, the promotion and standardization of autotransplantation technology will be further enhanced, ultimately benefiting a broad range of patients. Although research on digestive system autotransplantation is relatively in-depth, quantitative data is still lacking. Despite some scholars discussing studies with conflicting data through meta-analyses, the depth of discussion is relatively limited and subjective (32–34). Moreover, many scholars' research on digestive system autotransplantation remains at the level of literature review and personal clinical experience summaries (9, 35).

Bibliometrics is a tool for quantitatively analyzing literature, which can reveal research hotspots, frontier dynamics, and research trends in a particular field (36). Through statistical analysis of relevant literature, we can understand the research activity, main contributors, and collaboration relationships among research institutions in the field, thereby providing a scientific basis for future research. Bibliometrics, through quantitative analysis of a large amount of literature data, helps researchers identify and evaluate scientific research trends and patterns. It typically involves several analyses: counting publications over time to gauge research activity and trends; identifying research hotspots through keyword and subject term co-occurrence; evaluating impact via citation analysis; and examining collaboration networks to understand relationships among research institutions and authors. Through these analyses, we can systematically review the research status of the field of digestive system autotransplantation, identify key researchers and institutions, understand research hotspots and trends. This comprehensive overview will provide valuable insights for future research directions and promote better planning and collaboration within the scientific community.

In this study, we aim to systematically review and identify the key areas of focus and emerging patterns in the field of digestive system autotransplantation. Using visualization tools VOSviewer and CiteSpace, we reviewed and analyzed relevant literature from 2004 to 2024. We conducted a systematic analysis of the current state and evolving patterns in digestive system autotransplantation using bibliometric methods. Through these analyses, we can systematically review the research status of the field of digestive system autotransplantation, identify key researchers and institutions, understand research hotspots and trends. This comprehensive analysis will offer important perspectives for guiding future research directions and promote better planning and collaboration within the scientific community.

## Methods and materials

The Web of Science Core Collection (WoSCC) database is known for its precise document type labeling, making it ideal for literature analysis. Thus, we selected this database for our search (37, 38). On May 22, 2024, we conducted a search in WoS for articles on digestive system autotransplantation published between 2004 and 2024. To ensure comprehensive coverage and minimize the risk of missing relevant literature, the search strategy incorporated a wide range of synonyms and related terms for “autotransplantation” and the specific digestive organs. The detailed search strategy, including the combination of keywords and operators used, is presented in Supplementary Table S1. The search formula included: ((((((((TS=(Autotransplantation)) OR TS=(“Autologous Transplantation”)) OR TS=(“Autologous Transplantations”)) OR TS=(“Transplantations, Autologous”)) OR TS=(Autografting)) OR TS=(Autograftings)) OR TS=(Autotransplantation)) OR TS=(Autotransplantations) AND ((((((((((TS=(Liver)) OR TS=(Livers)) OR TS=(Pancreas)) OR TS=(pancreatic)) OR TS=(“Intestine, Small”)) OR TS=(“Intestines, Small”)) OR TS=(“Small Intestines”)) OR TS=(“Small Intestine”))) OR TS=(intestine)) OR (((((((((((TS=(“autologous liver transplant”)) OR TS=(“autologous liver transplantation”)) OR TS=(“Autologous Pancreas Transplantation”)) OR TS=(“Autologous Pancreas Transplant”)) OR TS=(“autologous small intestine transplant”)) OR TS=(“Autologous small bowel transplantation”)) OR TS=(“intestinal autotransplantation”)) OR TS=(“Pancreas autotransplantation”)) OR TS=(“intestinal autotransplant”)) OR TS=(“Pancreas autotransplant”)) OR TS=(“Liver autotransplantation”)) OR TS=(“liver autotransplant”).

The criteria for selecting literature for this study were: (1) full-text publications on digestive system autotransplantation; (2) articles and reviews in English; and (3) publications from 2004 to 2024. Exclusion criteria included: (1) unrelated topics; and (2) conference abstracts, news, brief reports, etc. Plain text versions of the papers were extracted.

**Figure 1:**
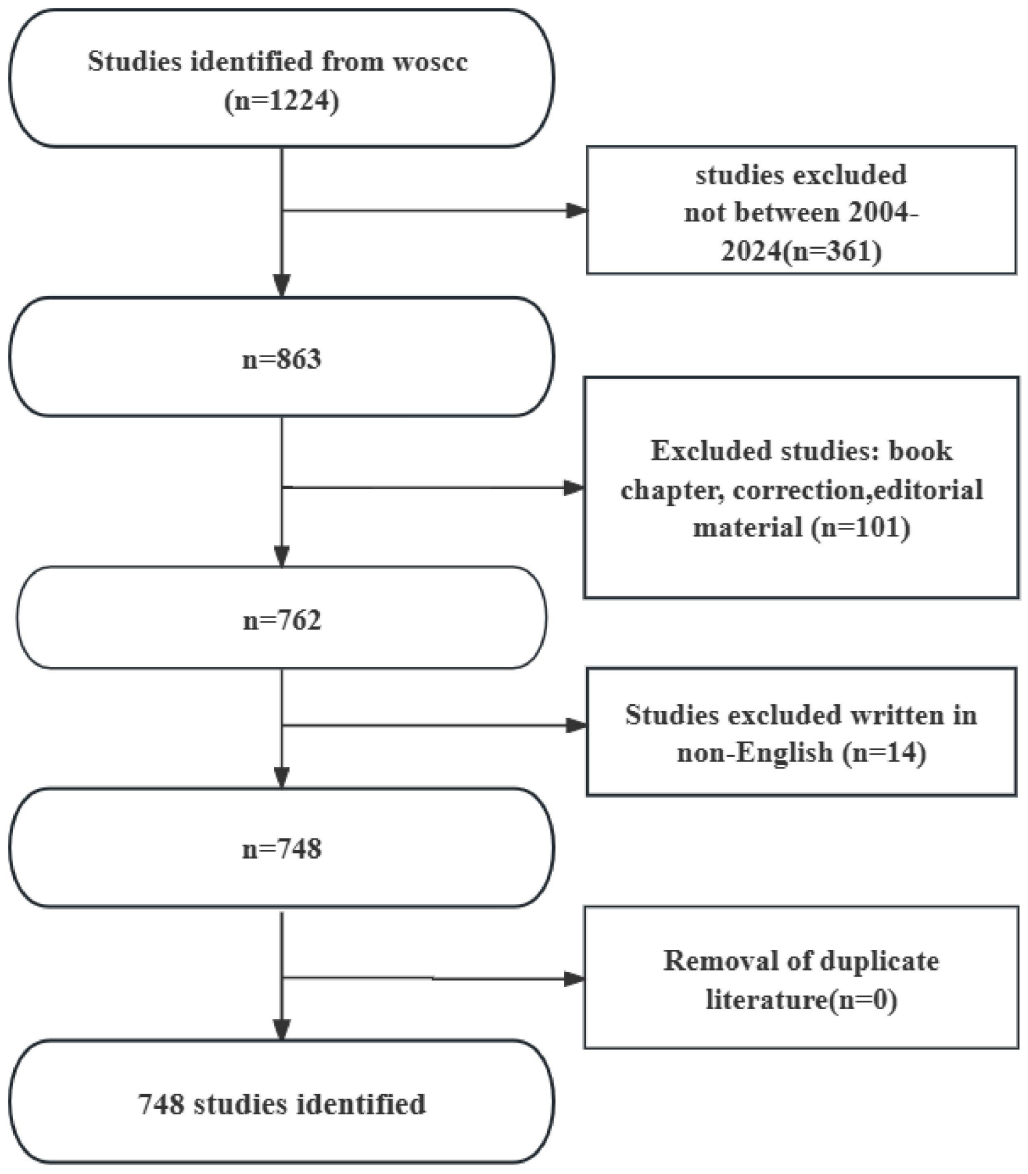
Flowchart of this study.

We utilized GraphPad Prism v8.0.2 for analyzing and plotting annual and national publication trends and proportions. Additionally, CiteSpace (v6.2.4R) and VOSviewer (v1.6.18) were employed to analyze the data and create visual representations of the scientific knowledge maps. Figure 1 shows the flow chart of the literature selection process.

## Results

From 2004 to 2024, the WoSCC database recorded a total of 748 publications on autologous transplantation in the digestive system, comprising 618 research articles and 130 reviews. These publications involved 49 different countries, 943 institutions, and 3,822 authors.

**Figure 2:**
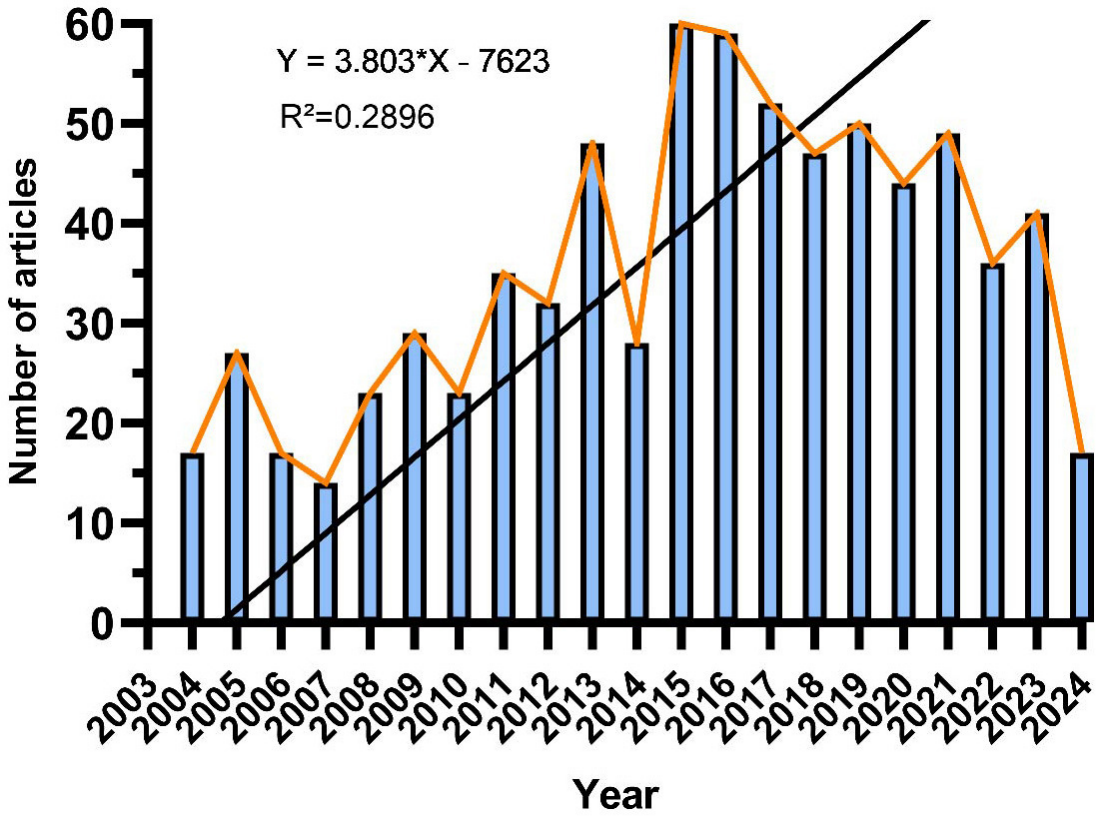
The trend in annual publication numbers on the application of digestive system autotransplantation from 2004 to 2024.

Since 2004, the annual number of published papers has shown a gradual rise (see Figure 2). This trend can be segmented into three phases: between 2004 and 2007, the yearly publication count was around 20, indicating slow growth and limited research interest; from 2008 to 2015, there was a steady increase in publications, peaking in 2015; post-2016, the number of publications started to decline gradually.

### Countries and institutions

**Figure 3:**
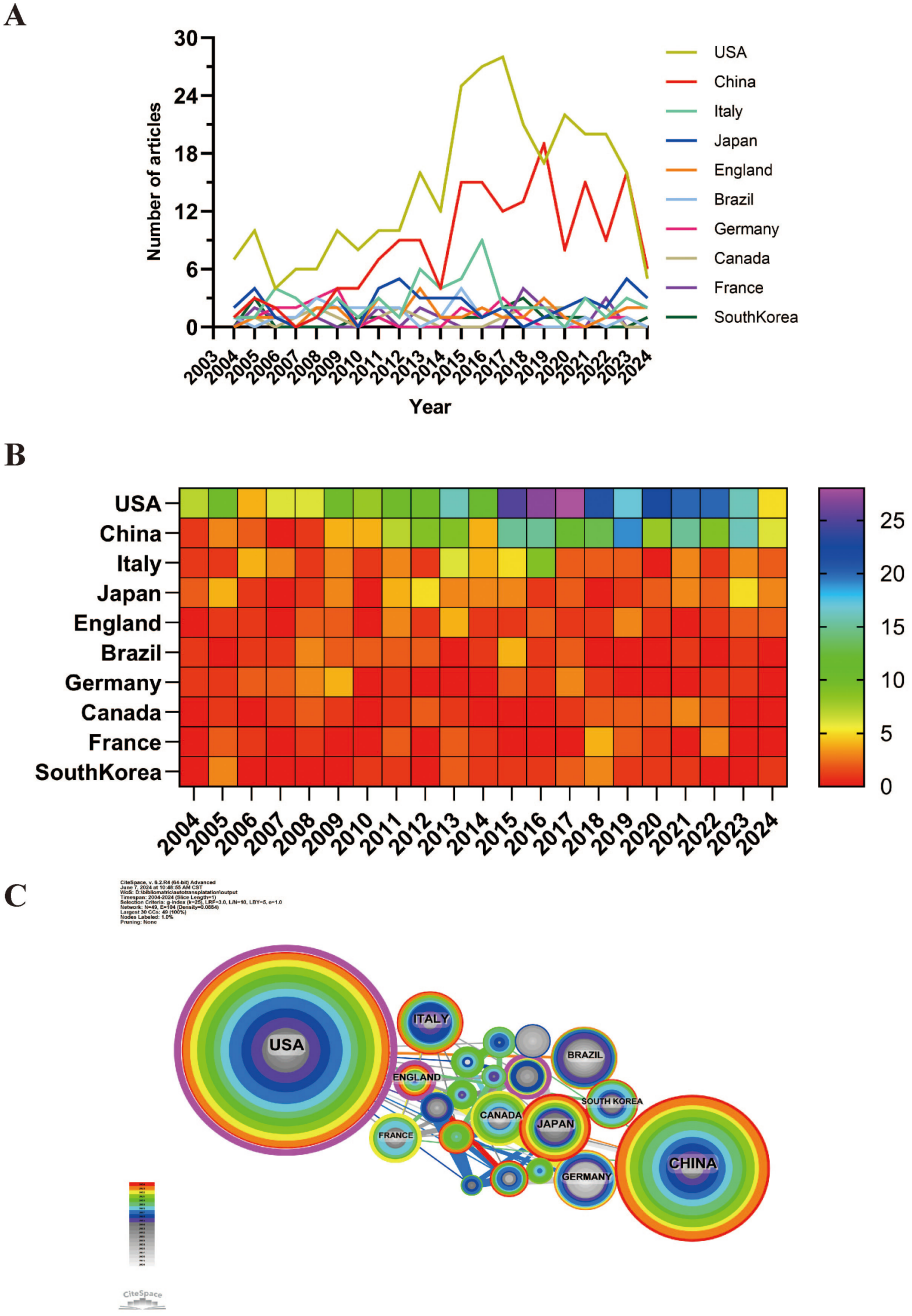
Over the past 20 years, the volume of publications and collaboration scenarios on the use of digestive system autotransplantation across different countries. **(A)** Publication Volume Line Chart; **(B)** heat map of publication volume; **(C)** International Collaboration Network Map.

Research on autologous transplantation in the digestive system spans 49 countries. Figures 3A, B illustrate the annual publication volume for the top 10 countries over the past decade. The leading five countries in this field are the United States, China, Italy, Japan, and the United Kingdom. The United States is the frontrunner, accounting for 40.11% of the total publications, significantly outpacing other countries.

Among the top 10 countries for paper publications, the United States stands out with a citation count of 7,323 (see Supplementary Table S2), far surpassing other nations. Additionally, its citation-to-publication ratio is 24.41, ranking sixth overall, which indicates the high quality of its publications. China is second with 172 publications and 2,561 citations, but its citation-to-publication ratio is relatively low at 14.89. Figure 3C depicts the collaboration network, showing close cooperation between the United States and China. The U.S. also collaborates extensively with Italy, the United Kingdom, and France, while China has strong partnerships with Japan, Germany, and South Korea. The United States not only excels in publication volume and citation frequency but also has a centrality score of 0.54, underscoring its leading role in the field.

**Figure 4:**
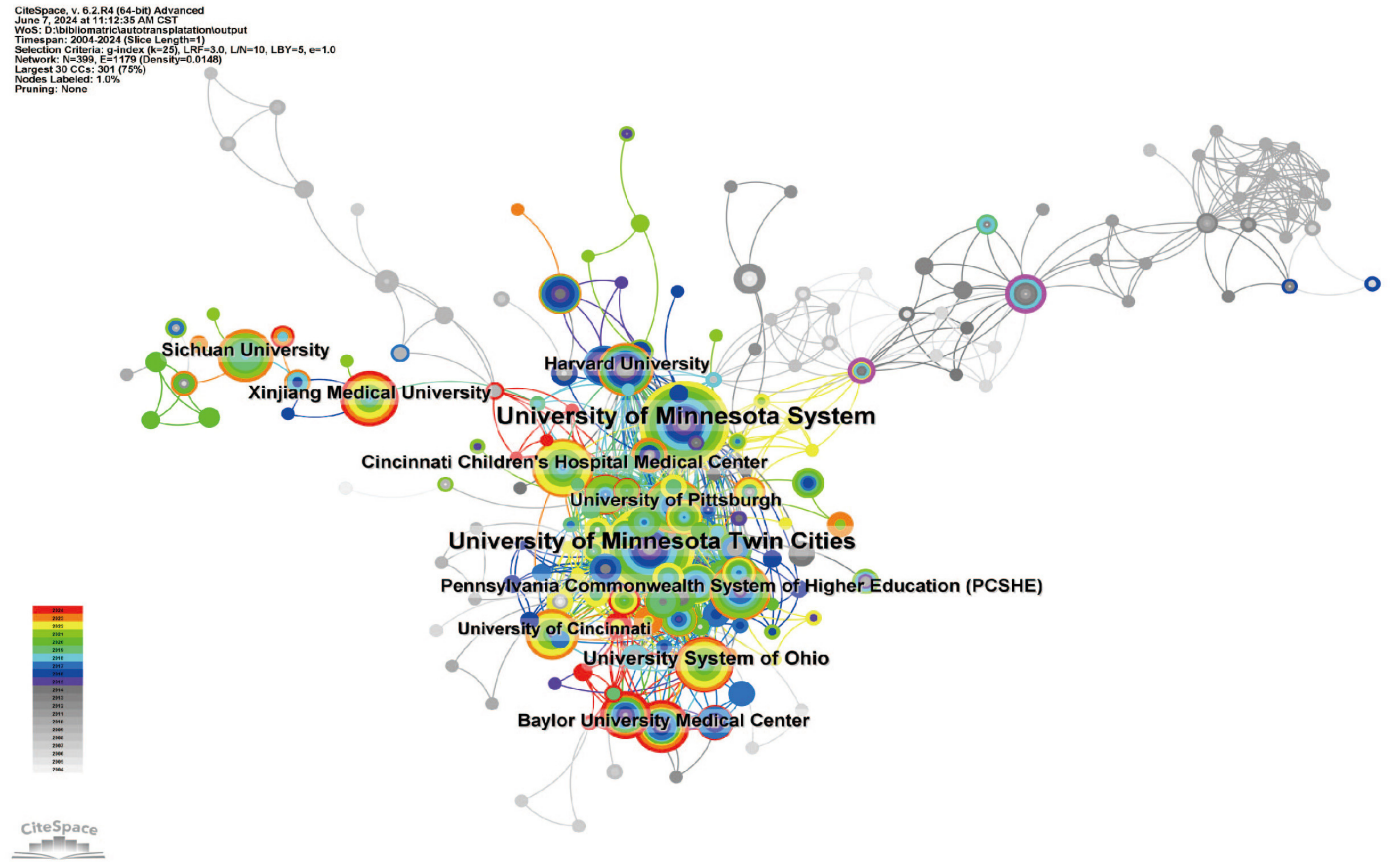
Network diagram illustrating institutional collaboration on the application of digestive system autotransplantation.

In total, 943 institutions have published articles on gastrointestinal autotransplantation. Of the top ten institutions by publication volume, eight are based in the United States, and two are located in China (refer to Supplementary Table S3 and Figure 4). The University of Minnesota System has the highest number of publications with 71 papers, cited 2,258 times, averaging 31.80 citations per paper. The University of Minnesota Twin Cities ranks second with 70 papers, cited 2,101 times, averaging 30.01 citations per paper. The University System of Ohio ranks third with 31 papers, cited 350 times, averaging 11.29 citations per paper. Cincinnati Children's Hospital Medical Center is ranked fourth, with 23 papers cited 227 times, averaging 9.87 citations per paper. Further analysis shows that institutions, both domestic and international, tend to collaborate more with others within their own countries. Therefore, we encourage increased collaboration between domestic and international institutions to break down academic barriers.

### Journal analysis and co-citation data

**Figure 5:**
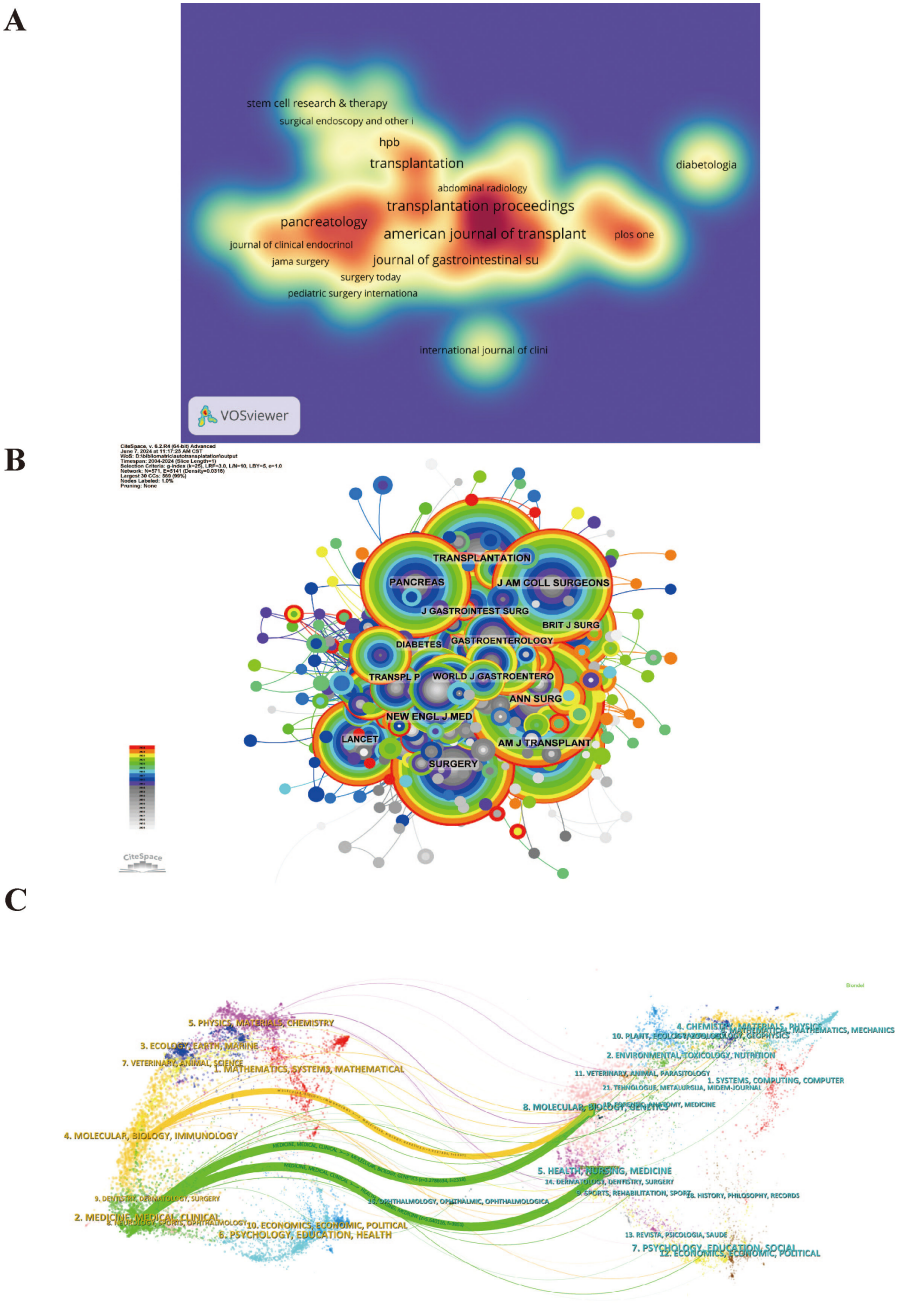
Information on journals publishing research on the application of digestive system autotransplantation and their co-cited journal data. **(A)** Journal publication density map; **(B)** Journal Co-citation Network Map; **(C)** Journal Dual Overlay Map (Left: citing journals. Right: cited journals).

Supplementary Tables S4, S5 detail the top 10 journals by publication volume and citation counts. The American Journal of Transplantation (30 papers, 4.01%) is the leading journal in this field, followed by Transplantation Proceedings (30 papers, 4.01%), and Transplantation (19 papers, 2.54%; Figure 5A). Of the top 10 prolific journals, Annals of Surgery boasts the highest impact factor (IF) of 10.1. Notably, 70% of these journals fall within the Q1 or Q2 categories.

A journal's influence is measured by the frequency of its co-citations, reflecting its importance within the scientific community. As shown in Figure 5B and Supplementary Table S5, the most frequently co-cited journal is “Annals of Surgery” (362 times), followed by “Transplantation” (338 times) and “Surgery” (300 times). Among the 10 most frequently co-cited journals, the “New England Journal of Medicine” stands out with 258 citations and the highest impact factor of 158.5. Notably, 80% of the co-cited journals fall within the Q1/Q2 quartiles.

The thematic distribution of academic publications is depicted using dual-map overlays (Figure 5C). Colored paths illustrate citation relationships, with citing journals on the left and cited journals on the right. The results reveal two primary citation paths: research published in journals related to molecular/biology/genetics is mainly cited by journals in molecular/biology/immunology and medicine/medical/clinical fields. Additionally, research published in journals related to health/nursing/medicine is primarily cited by journals in the medicine/medical/clinical fields.

**Figure 6:**
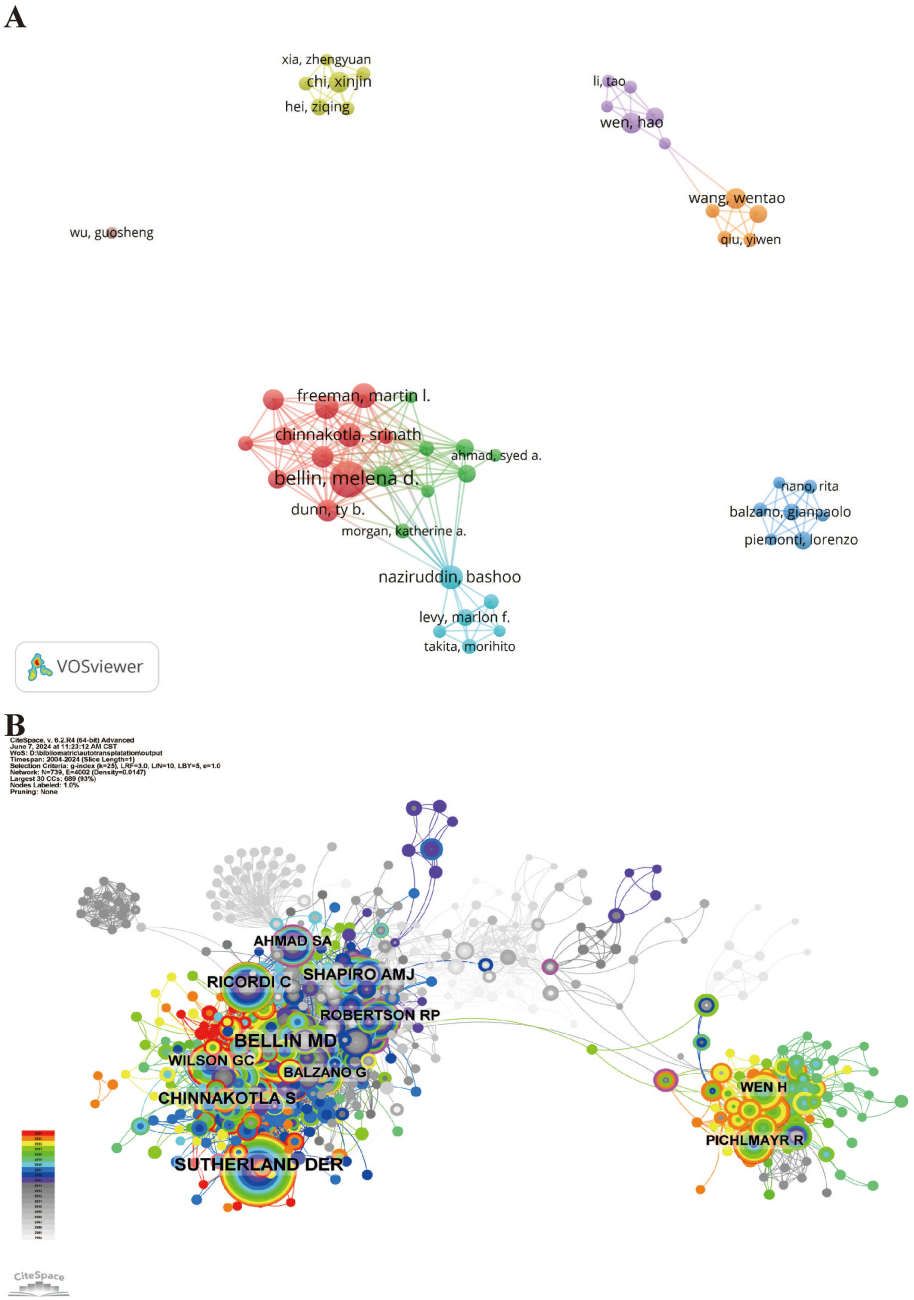
Author collaboration network map and author co-citation network map. **(A)** Author Collaboration Network Map; **(B)** Author Co-citation Network Map.

Among all authors who have published literature on digestive system autotransplantation, Supplementary Table S6 lists the top 10 authors with the highest number of publications. These leading authors have collectively contributed 223 papers, accounting for 29.81% of all publications in this field. Bellin, Melena D., stands out with the most research papers at 43, followed by Freeman, Martin L., with 23 papers. Chinnakotla, Srinath, Naziruddin, and Pruett, Timothy L. each have published 22 papers. CiteSpace provides a visualization of the collaborative network among these authors (Figure 6A).

Figure 6B and Supplementary Table S6 show the top 10 most co-cited and cited authors. In total, 146 authors have been cited more than 50 times, indicating the substantial influence and reputation of their research. The most prominent nodes in the co-citation network are Bellin, Melena D. (171 citations), Sutherland, D.E.R. (163 citations), and Chinnakotla, Srinath (100 citations). Further analysis reveals that Bellin, Melena D. ranks first in both the number of publications and citations, underscoring his significant impact and leadership in the field of digestive system autotransplantation.

These findings highlight the contributions of key researchers and their collaborative networks, providing insights into the influential figures and their connections within the field. The data suggests that a small group of prolific authors and highly-cited researchers drives much of the advancement in digestive system autotransplantation, indicating potential focal points for future research collaborations and studies.

### Co-cited references

**Figure 7:**
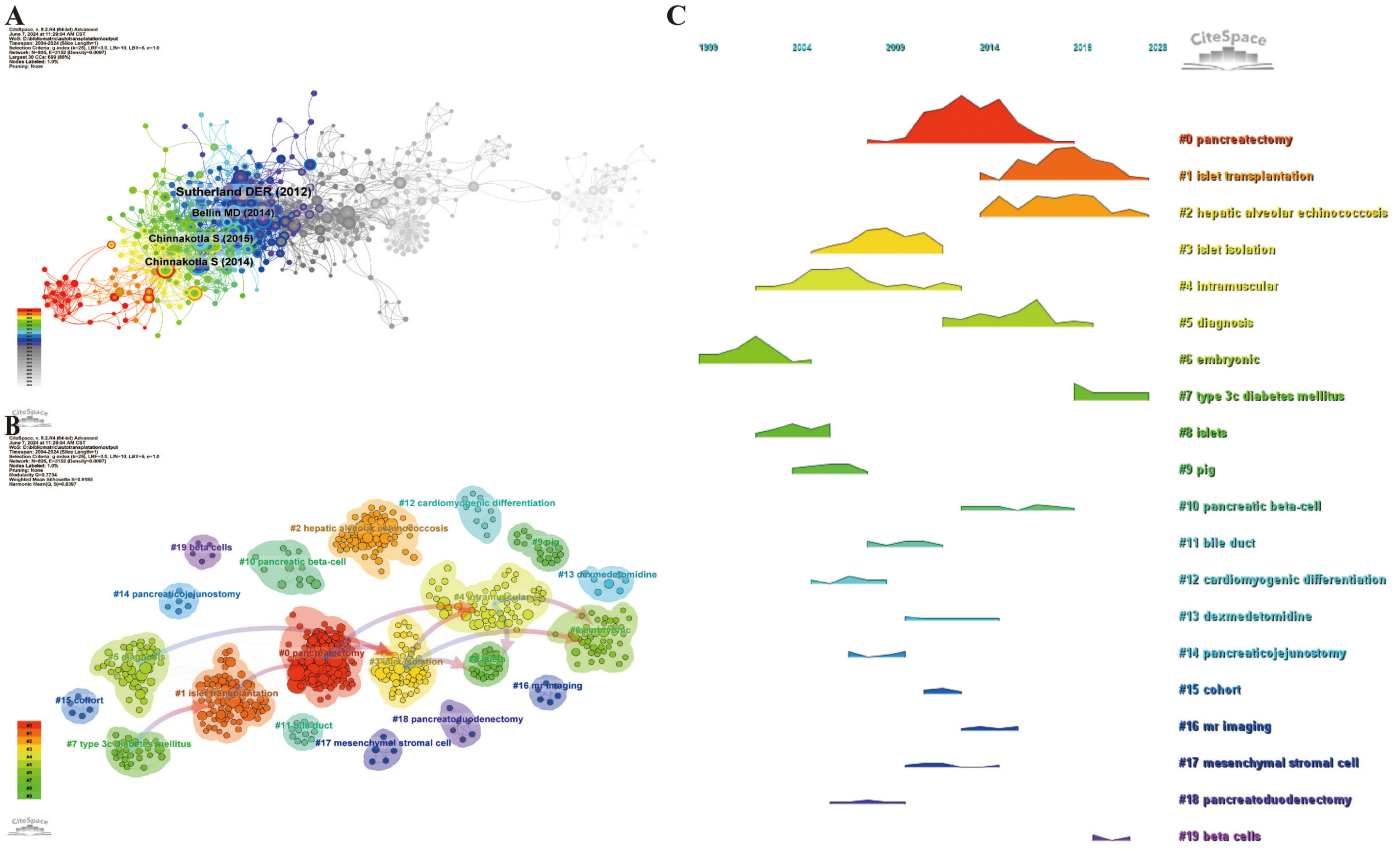
Co-cited reference information. **(A)** Co-cited Literature Network Map; **(B)** Co-cited Literature Clustering Map; **(C)** Co-cited Literature Volcano Plot.

The co-cited reference network, constructed using 1-year intervals from 2004 to 2024, consists of 805 nodes and 3,152 links (Figure 7A). Based on the top 10 most co-cited articles (Supplementary Table S7), the study “Total Pancreatectomy and Islet Autotransplantation for Chronic Pancreatitis,” published in the Journal of the American College of Surgeons, reports that total pancreatectomy (TP) combined with intraportal islet autotransplantation (IAT) can alleviate pain and preserve a significant number of β-cells in patients with chronic pancreatitis (CP) who are unresponsive to other treatments.

From February 1977 to September 2011, 49 CP patients, including 53 children aged 5–18 years, underwent TP-IAT. The causes of CP were as follows: idiopathic (41%), sphincter of Oddi dysfunction/biliary (9%), genetic factors (14%), pancreas divisum (17%), alcohol (7%), and other causes (12%). The average age was 35.3 years, with 74% of the patients being female. Prior surgeries included Puestow (9%), Whipple (6%), distal pancreatectomy (7%), and other surgeries (2%). Post-surgery, islet function was categorized into three groups: insulin-independent for those not using insulin, partial islet function for those with hyperglycemia managed by daily insulin or C-peptide positive, and insulin-dependent for those requiring standard insulin therapy.

Starting in 2007, patients completed the SF-36 quality of life survey before treatment and during continuous follow-ups, with an additional comprehensive survey added in 2008. The actuarial survival rates post TP-IAT were 96% for adults and 98% for children at 1 year, and 89% and 98% at 5 years, respectively. Complications requiring reoperation occurred in 15.9% of patients, with bleeding being the most common at 9.5%. IAT function was achieved in 90% of patients (C-peptide >0.6 ng/ml). After 3 years, 30% of patients were insulin-independent (25% of adults and 55% of children), and 33% had partial islet function. Additionally, 82% had an average HbA1c below 7.0%. Prior pancreatic surgeries were associated with reduced islet yield (2,712 vs. 4,077/kg; *p* = 0.003). Islet yield categories correlated with function: < 2,500/kg (36%), 2,501–5,000/kg (39%), >5,000/kg (24%), with corresponding 3-year insulin dependence rates of 12%, 22%, and 72%, and partial function rates of 33%, 62%, and 24%.

All patients reported experiencing pain before TP-IAT, with nearly all using narcotics daily. Post-treatment, 85% reported improved pain levels, and after 2 years, 59% had ceased using narcotics. Among children, 94% reported pain improvement, with 67% being pain-free, although 39% continued using narcotics at follow-up. SF-36 survey results showed significant improvement in all dimensions, both physical and mental, compared to baseline (*p* < 0.01). TP can effectively relieve pain and improve the quality of life for refractory CP patients, even if complete narcotic withdrawal is delayed or incomplete. IAT preserves islet function in most patients, with over two-thirds maintaining function and a quarter of adults and half of children achieving insulin independence.

The second most cited article, “Total Pancreatectomy and Islet Autotransplantation in Children for Chronic Pancreatitis: indications, surgical techniques, postoperative management, and long-term outcomes,” published in Annals of Surgery by Chinnakotla, Srinath, details the surgical techniques, complications, and long-term outcomes of TP-IAT. Surgical treatment for pediatric pancreatitis remains challenging; while partial resection or drainage surgery often provides temporary pain relief, long-term recurrence is common due to diffuse pancreatic involvement. TP eliminates the pain source, and IAT potentially prevents or mitigates TP-related diabetes.

A retrospective study conducted from 1989 to 2012 reviewed 75 children with CP who underwent TP-IAT after failing medical, endoscopic, or previous surgical treatments. Following TP-IAT, 90% of patients showed significant improvement in pancreatitis pain and severity (*p* < 0.001), with sustained narcotic relief. Of the 75 patients, 31 (41.3%) achieved insulin independence. Factors associated with insulin independence included younger age (*p* = 0.032), absence of prior Puestow procedure (*p* = 0.018), smaller body surface area (*p* = 0.048), higher islet equivalents per kilogram (IEQ; *p* = 0.001), and a total IEQ >100,000 (*p* = 0.004).

Multivariable analysis identified three significant factors associated with post-TP-IAT insulin independence: male gender, smaller body surface area, and higher total IEQ per kilogram. The total IEQ >100,000 was the single most significant factor associated with insulin independence (odds ratio = 2.62; *p* < 0.001). β-cell function was dependent on islet yield. The study concludes that TP-IAT is an effective treatment for children with painful pancreatitis unresponsive to medical and/or endoscopic treatments, providing significant long-term pain relief and improving quality of life.

We performed co-cited reference clustering and temporal clustering analysis, as shown in Figures 7B, C. Our findings reveal that intramuscular (cluster 4), embryonic (cluster 6), islets (cluster 8), and pig (cluster 9) were early research hotspots. Mid-period research hotspots included islet isolation (cluster 3), bile duct (cluster 11), cardiomyogenic differentiation (cluster 12), dexmedetomidine (cluster 13), pancreaticojejunostomy (cluster 14), cohort (cluster 15), MR imaging (cluster 16), mesenchymal stromal cell (cluster 17), and pancreatoduodenectomy (cluster 18). Current and trending topics in the field are pancreatectomy (cluster 0), islet transplantation (cluster 1), hepatic alveolar echinococcosis (cluster 2), diagnosis (cluster 5), type 3c diabetes mellitus (cluster 7), and beta cells (cluster 19).

**Figure 8:**
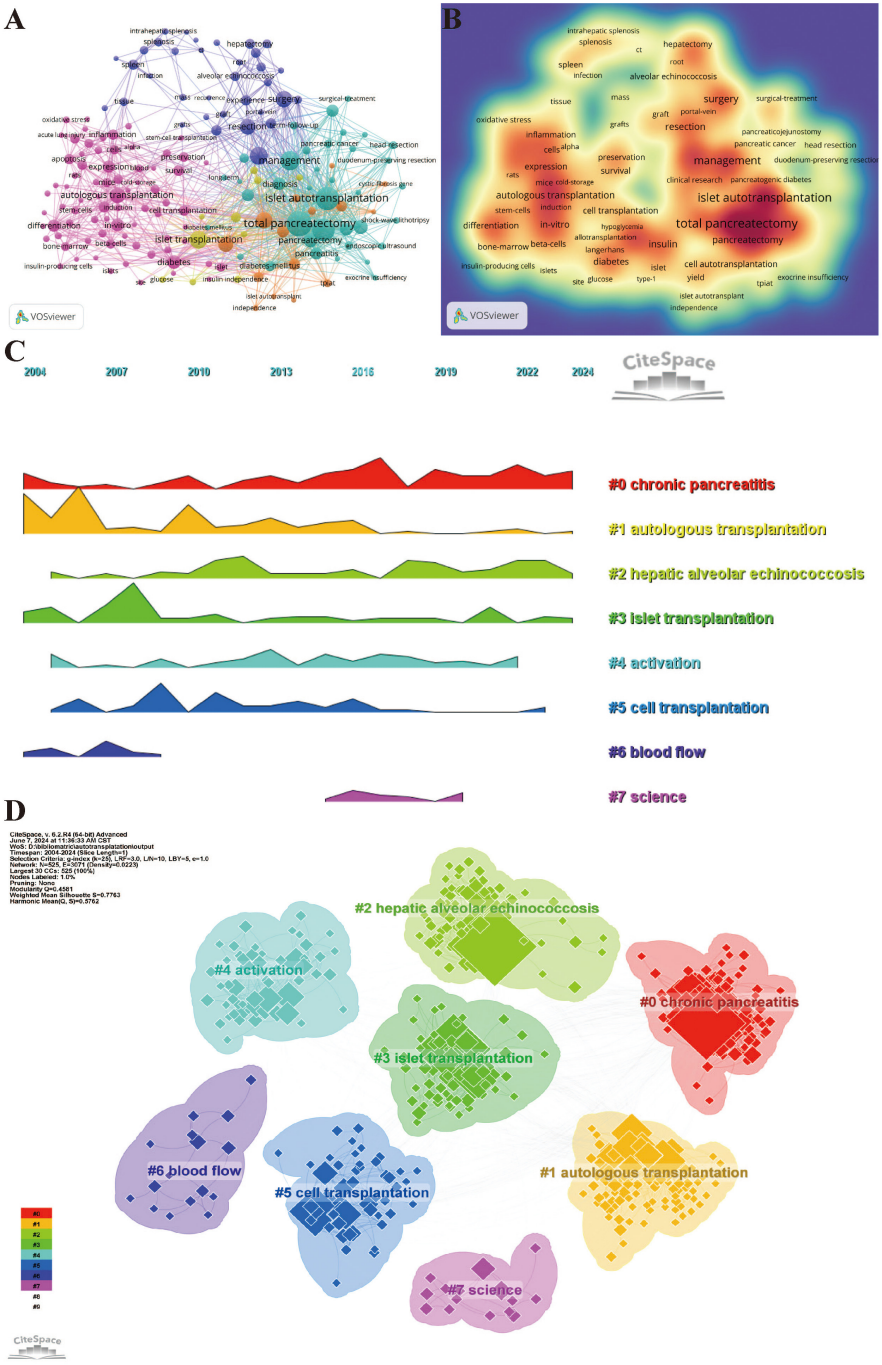
Keyword analysis of articles on the application of digestive system autotransplantation. **(A)** High-Frequency Keywords Network Map; **(B)** Keyword Density Map; **(C)** Keyword Clustering Volcano Plot; **(D)** Keyword Clustering Map.

Using VOSviewer to examine keyword co-occurrence, we identified the most frequently used keywords: “complications” (163 occurrences), among others (Supplementary Table S8, Figures 8A, B). We excluded unrelated keywords and built a network of 172 keywords that appeared at least seven times, resulting in five distinct clusters. Cluster 1 (pink) contains 76 keywords including autologous transplantation, angiogenesis, stem cell, diabetes, cell transplantation, survival, preservation, blood, T cell, regeneration, apoptosis, oxidative stress, reperfusion injury, inflammation, beta cells, bone marrow, cold storage, differentiation, donor, failure, gene therapy, ischemia reperfusion injury, pig, model, pancreatic islets, and pathogenesis. Cluster 2 (green) includes 37 keywords such as total pancreatectomy, pain, chronic pancreatitis, quality of life, islet autotransplantation, long-term outcome, exocrine insufficiency, efficacy, follow-up, management, multicenter, and head resection. Cluster 3 (purple) comprises 34 keywords including outcome, surgery, complication, resection, experience, hepatectomy, tumor, infection, graft, impact, mass, inferior vena cava, reconstruction, root, splenectomy, tissue, and implantation. Cluster 4 (yellow) contains 15 keywords such as acute autotransplantation, children, chronic pancreatitis, cystic fibrosis gene, independence, insulin, risk, mutation, total pancreatectomy with islet autotransplantation (TPIAT), yield, and hereditary pancreatitis. Cluster 5 (yellow) consists of 10 keywords including beta cell function, clinical research, glucose, insulin independence, islet isolation, islets of Langerhans, pediatrics, and practice. We used CiteSpace to create a volcano plot that visually displays how research hotspots have evolved over time (Figures 8C, D).

### Co-cited references and keywords

**Figure 9:**
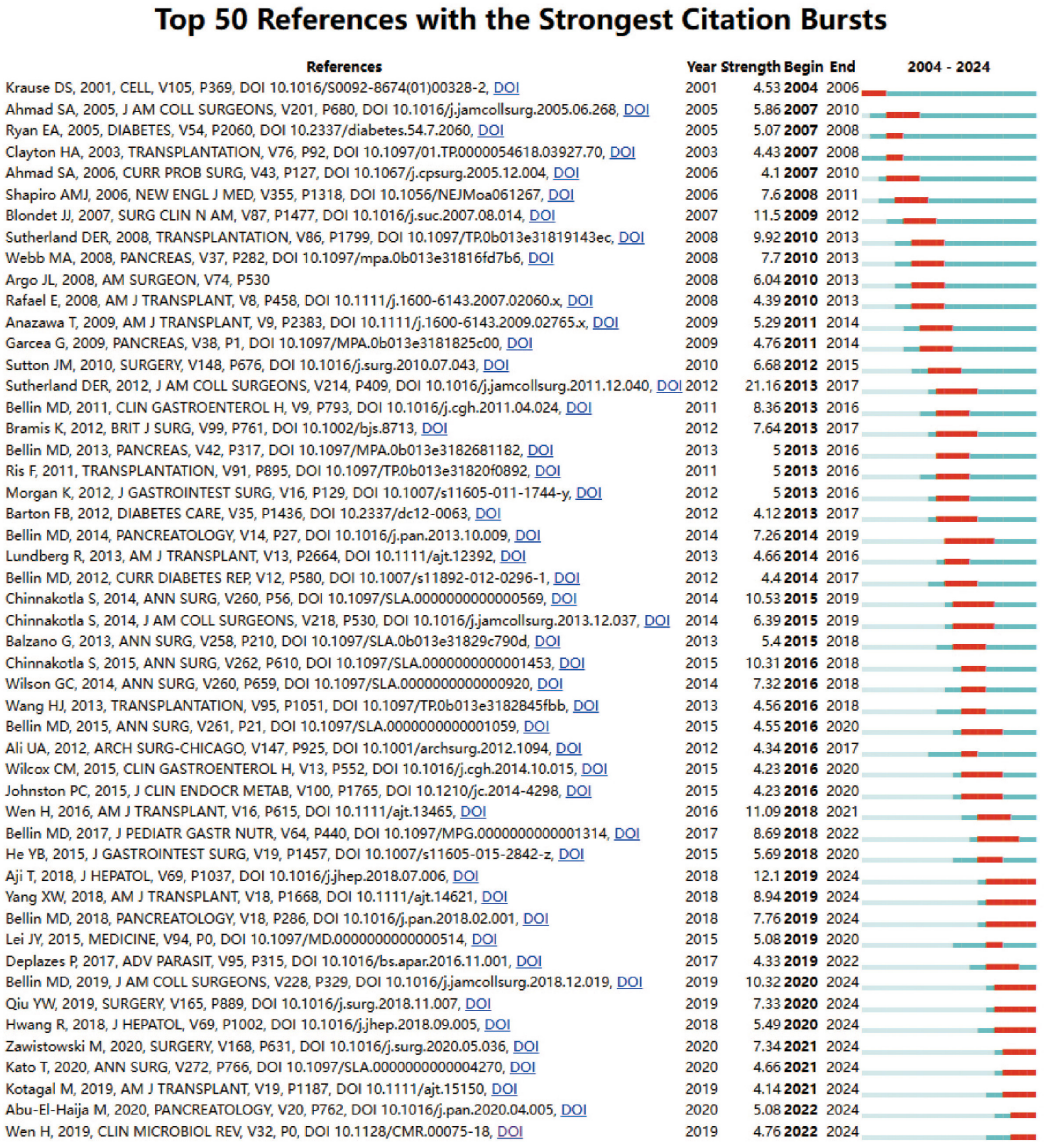
Top 50 references with the strongest citation bursts.

Using CiteSpace, we identified the 50 most reliable citation bursts in the field of digestive system autotransplantation. All 50 references were published between 2004 and 2024, indicating their frequent citation over the past two decades. Importantly, 12 of these papers are currently at their peak citation period (Figure 9), suggesting that digestive system autotransplantation will continue to be a focal point in future research.

**Figure 10:**
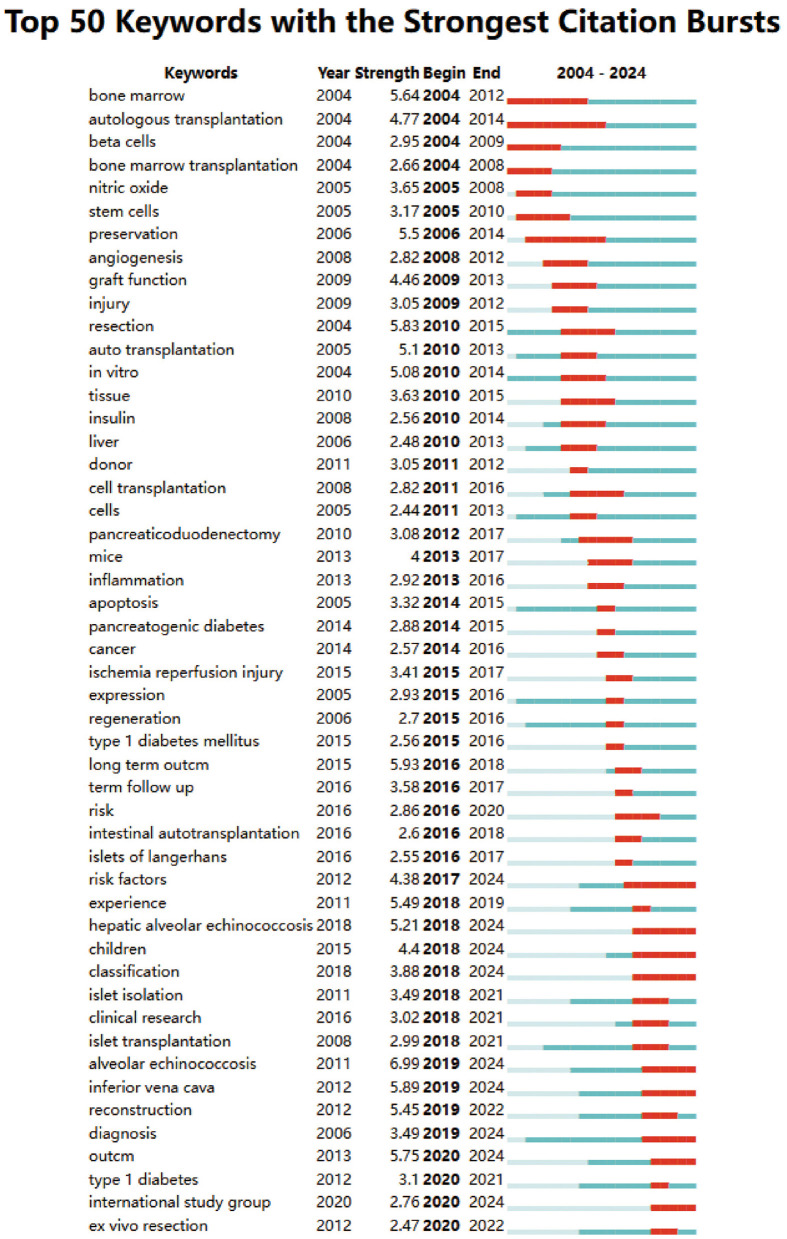
Top 50 keywords with the strongest citation bursts.

Among the 576 strongest burst keywords in this field, we focused on the top 50 with the highest burst strength (Figure 10). These keywords represent current research hotspots and potential future directions in the field.

## Discussion

This study systematically retrieved and analyzed literature from the WoSCC database on autologous transplantation of the digestive system, comprehensively showcasing the research trends and academic influence in this field over the past two decades. Autologous transplantation of the digestive system is a significant surgical technique, particularly crucial in treating hepatic hydatid disease, chronic pancreatitis, and certain refractory digestive system diseases. This systematic review of the literature in this field helps understand its current research status, hotspots, and future development directions. The primary reason for selecting the WoSCC database for literature retrieval is its high accuracy in documenting literature types, providing comprehensive and detailed records. By retrieving relevant literature from 2004 to 2024, we ensured the completeness and timeliness of the data. The search strategy employed multiple keywords and synonyms to cover all relevant literature. The establishment of inclusion and exclusion criteria further enhanced the scientificity and rationality of literature screening, ensuring the quality and reliability of the data.

From the perspective of publication trends, since 2004, the number of publications on autologous transplantation of the digestive system has generally shown an upward trend, peaking between 2008 and 2015. This growth trend may reflect the continuous development of new technologies and treatment methods during this period. However, since 2016, the number of publications has declined, possibly due to a shift in research focus, the maturation of new technologies, and competition from other treatment methods. This change in trend suggests the need to pay attention to emerging hotspots and potential future development directions in this field.

Although the number of publications has decreased in recent years, it does not imply a reduction in the importance of autologous transplantation techniques for the digestive system. On the contrary, these techniques continue to play an indispensable role in clinical applications, providing solutions that other treatment methods cannot replace, especially in handling complex cases and improving patient survival quality. Therefore, future research should continue to focus on improving the operability and safety of these techniques, optimizing postoperative management strategies, and exploring new application scenarios to ensure patients receive the best treatment outcomes and quality of life.

Autologous liver transplantation is a crucial surgical technique, particularly suitable for patients who cannot undergo conventional liver transplantation. This technique is primarily used to treat diseases such as primary liver cancer, hepatic hydatid disease, and hepatic hemangioma (39, 40). A significant application of autologous liver transplantation involves liver resection, followed by *ex vivo* liver repair and subsequent reimplantation into the patient. Literature shows that autologous liver transplantation significantly improves patient survival rates and quality of life. However, the technique is complex, and the management of postoperative complications and long-term follow-up remain essential research directions.

Autologous pancreatic transplantation, mainly total pancreatectomy with islet autotransplantation (TP-IAT), plays a crucial role in treating chronic pancreatitis (41). This technique involves removing the diseased pancreas and transplanting healthy islet cells back into the patient, aiming to alleviate pain and preserve endocrine function. Studies indicate that TP-IAT not only significantly reduces patient pain but also enhances insulin independence and improves quality of life. However, improving the survival rate and function of islet cells remains a primary research focus. Additionally, research has shown that the effectiveness of TP-IAT is closely related to factors such as the patient's age and the number of islets, necessitating further in-depth studies (42).

Autologous small intestine transplantation offers unique advantages in treating small intestine diseases and short bowel syndrome. This technique involves resecting the diseased small intestine and reimplanting healthy segments to restore normal small intestine function. Research indicates that autologous small intestine transplantation significantly improves nutrient absorption and reduces dependence on parenteral nutrition. However, the technique is challenging, and managing postoperative complications (such as graft dysfunction and infections) remains an important research direction. In recent years, advancements in surgical techniques and postoperative management have gradually increased the success rate of autologous small intestine transplantation, but further research is needed to optimize surgical protocols and enhance long-term survival rates.

From a national perspective, the United States has made the most significant contributions to research in this field, with the highest number of publications and citations, reflecting its leading position in autologous transplantation research for the digestive system. China, as the second-largest contributor, has seen a significant increase in research in recent years, demonstrating strong development momentum. Notably, a portion of China's research output addresses conditions like hepatic alveolar echinococcosis (HAE) (43), a parasitic disease endemic in certain regions where autologous liver transplantation offers a curative option for complex cases (44). Our analysis identified HAE as a current research hotspot (Cluster 2 in co-cited reference clustering), and Chinese institutions contribute to the literature in this specific area. While a detailed comparative analysis of Sino-US research on HAE is beyond the scope of this bibliometric study, the data suggest China's growing expertise in applying autotransplantation techniques to challenging regional diseases (45).

Italy, Japan, and the United Kingdom also have considerable influence in this field, with high publication volumes and citation rates reflecting their important roles in digestive system autologous transplantation research.

From an institutional perspective, the University of Minnesota System and the University of Minnesota Twin Cities are the institutions with the highest number of publications in this field, indicating their significant influence in digestive system autologous transplantation research. Analysis shows that these leading institutions not only have a high number of publications but also a high citation count of their research outcomes, reflecting widespread academic recognition and impact. Collaboration between domestic and international institutions shows that while domestic institutions collaborate closely, cross-national collaborations, especially between China and the United States, play a crucial role in advancing research in this field.

From a journal perspective, the American Journal of Transplantation and Transplantation Proceedings are the journals with the most publications, indicating their important position in digestive system autologous transplantation research. High-impact factor journals have significant authority and influence in this field, with Annals of Surgery and Transplantation being the most co-cited journals, further corroborating their core status in digestive system autologous transplantation research. These journals not only produce a high volume of research but also high-quality studies, representing the forefront and hotspots of this field.

Among all authors who have published literature on digestive system autologous transplantation, Bellin, Melena D., and Freeman, Martin L. are the most prolific. The research outcomes of these high-output authors have significantly contributed to the development of this field. Analysis indicates that these authors not only dominate in terms of publication volume but their research outcomes are also highly cited, reflecting their influence in the academic community. Further analysis of the author collaboration network shows that research in the field of digestive system autologous transplantation is highly collaborative, especially among prolific authors, which helps to advance the depth and breadth of research.

Through keyword analysis, we can identify the main hotspots in digestive system autologous transplantation research, including total pancreatectomy, islet autotransplantation, and chronic pancreatitis. The network diagram of high-frequency keywords shows close associations between different keywords, representing the main research directions and focal points in this field.

The co-occurrence analysis of keywords reveals several major clusters in this field of research: first, studies on autologous transplantation and related treatment methods; second, studies on pancreatic transplantation, its associated complications, and management; third, studies on surgical techniques and clinical outcomes. For instance, Cluster 1 (pink), which includes terms such as “autologous transplantation,” “stem cell,” “angiogenesis,” “regeneration,” “oxidative stress,” “reperfusion injury,” and “inflammation,” points to fundamental biological processes and therapeutic strategies. “Stem cells,” “angiogenesis,” and “regeneration” reflect the ongoing research into enhancing organ repair and functional recovery post-transplantation. Concurrently, keywords like “oxidative stress,” “reperfusion injury,” and “inflammation” underscore critical challenges in transplantation, as these processes can significantly impact graft viability and patient outcomes; research in these areas aims to develop strategies for their mitigation. Cluster 2 (green), featuring “total pancreatectomy,” “pain,” “chronic pancreatitis,” and “quality of life,” directly relates to the primary clinical applications and patient-centered outcomes in pancreatic autotransplantation, particularly for debilitating chronic pancreatitis. Cluster 3 (purple), with keywords like “outcome,” “surgery,” “complication,” “resection,” and “tumor,” encompasses the surgical aspects, oncological indications, and overall results of these complex procedures across different organs. Clusters 4 (yellow) and 5 (yellow), focusing on “TPIAT,” “children,” “insulin independence,” “islet isolation,” and “beta cell function,” highlight the specialized area of islet autotransplantation, especially in pediatric populations and the efforts to preserve endocrine function. These research hotspots reflect the diversity and broad scope of research in digestive system autotransplantation.

Delving deeper into these hotspots, the prominence of total pancreatectomy with islet autotransplantation (TPIAT) for chronic pancreatitis is evident from its frequent appearance in keyword clusters (e.g., Cluster 2 and 4) and its focus in highly co-cited articles. TPIAT is significant because it offers a definitive solution for intractable pain in patients with end-stage chronic pancreatitis, a condition that severely impairs quality of life (46, 47). The simultaneous autotransplantation of islets aims to preserve endocrine function and prevent or mitigate brittle diabetes, a major drawback of total pancreatectomy alone (48). Current research in TPIAT, as suggested by keywords like “islet yield,” “insulin independence,” and “long-term outcome,” focuses on optimizing patient selection, improving islet isolation and engraftment techniques to maximize insulin independence, and understanding long-term metabolic and quality-of-life outcomes, particularly in diverse populations including children. Challenges remain in consistently achieving high islet yields and durable insulin independence, as well as managing exocrine pancreatic insufficiency post-surgery. Opportunities lie in refining islet processing, exploring adjuvant therapies to enhance islet survival (potentially linking to Cluster 1's themes of regeneration and cytoprotection), and standardizing postoperative management protocols.

The strong focus on “islet transplantation” itself, often in the context of TPIAT but also as a broader concept, underscores its critical role. Its significance lies in the potential to transform a life-altering surgery (total pancreatectomy) into a more manageable condition by preserving endocrine function. The challenges revolve around the viability and functionality of transplanted islets, with research opportunities focused on enhancing isolation techniques, improving islet culture and preservation, and finding ways to protect islets from immediate post-transplant loss and ensure long-term survival and function (again, connecting to “beta cells,” “inflammation,” and “oxidative stress” from Cluster 1).

Furthermore, “surgical techniques” and “complications” are consistently highlighted keywords (e.g., Cluster 3). The complexity of digestive system autotransplantation means that refinements in surgical approaches—such as *ex vivo* resection, vascular reconstruction, and organ preservation—are paramount for success. The significance of advancing these techniques is direct: improved patient safety, reduced operative morbidity, and the ability to tackle increasingly complex cases, such as extensive tumors or severe anatomical distortions. Prominent challenges include managing complex vascular work, minimizing ischemia-reperfusion injury (a key term in Cluster 1), and reducing operative times. Opportunities for advancement include the adoption of minimally invasive techniques where feasible, enhanced intraoperative imaging and navigation, 3D modeling for preoperative planning, and further research into optimal organ preservation solutions (49). The high frequency of “complications” underscores the ongoing need to better understand, prevent, and manage adverse events such as bleeding, infection, thrombosis, and graft dysfunction. This presents opportunities for developing predictive risk models, standardizing perioperative care, and innovating strategies to mitigate common complications, thereby improving overall outcomes and patient recovery.

Further analysis shows that research hotspots vary across different stages. In the early stages, research primarily focused on basic research and initial clinical applications, such as technical improvements in islet transplantation and pancreatectomy and preliminary efficacy evaluations. In the mid-stages, research hotspots gradually shifted to clinical applications and management, such as managing complications post-pancreatic transplantation and improving patients' quality of life. In the later stages, as technology matured and clinical applications deepened, research focus shifted more toward long-term outcomes and large-scale clinical trials, such as long-term survival rates, preservation of islet function, and long-term improvement in patients' quality of life.

Although this study systematically analyzed literature in the field of digestive system autologous transplantation, there are still some limitations. For example, although the WoSCC database is highly authoritative, it may still miss some relevant literature not included in the database. Additionally, bibliometric methods primarily rely on quantitative indicators such as publication volume and citation counts, potentially overlooking some significant qualitative research outcomes.

Reflecting on the dynamism of the field, our analysis of citation bursts (Figure 9) revealed that 12 key references are currently in their peak citation period, suggesting their topics represent active and impactful areas of ongoing research within the last few years. While a comprehensive review of all recent high-impact articles is extensive, these burst references often point toward refinements in surgical techniques, long-term outcome assessments, and management of specific complications.

Future research could further combine qualitative analysis methods to deeply explore the intrinsic logic and development paths of research in digestive system autologous transplantation. Moreover, with the continuous emergence of new technologies and methods, research hotspots and directions in this field may change, necessitating continuous attention to these changes and timely adjustments in research strategies. For instance, future research might need to focus more on personalized treatment plans based on artificial intelligence and big data, as well as the application of new biomaterials and regenerative medicine in autologous transplantation. Additionally, emerging technologies such as 3D printing for surgical planning and the creation of patient-specific anatomical models or guides are beginning to show promise in complex surgeries and could influence future approaches in autotransplantation, although they may not yet be dominant themes in the retrospective bibliometric data up to 2024. The development of advanced imaging and intraoperative navigation systems also continues to enhance surgical precision.

Furthermore, interdisciplinary research and international cooperation will be crucial in advancing this field. By integrating knowledge and technologies from different disciplines and strengthening international collaboration, research outcomes can be accelerated for translation into clinical applications, thereby improving the efficacy of digestive system autologous transplantation and the quality of life for patients.

## Conclusions

Our systematic analysis of the literature on autologous transplantation in the digestive system has uncovered the current state of research, key areas of interest, and evolving trends in this field. The findings demonstrate that the United States holds a dominant position, with significant contributions from countries like China. The primary research focuses are pancreatic transplantation, associated treatment methods, and clinical outcomes. To advance this field, future research should prioritize international collaboration and incorporate qualitative analysis techniques to deeply investigate the core aspects and developmental trajectories of related studies. This study not only maps the research landscape of autologous transplantation in the digestive system but also provides valuable references for future research. We hope this study will offer useful insights for researchers, thereby fostering the continued development and progress of autologous transplantation in the digestive system.

## Data Availability

The original contributions presented in the study are included in the article/Supplementary material, further inquiries can be directed to the corresponding authors.

## References

[1] Bellin MD Gelrud A Arreaza-Rubin G Dunn TB Humar A Morgan KA . Total pancreatectomy with islet autotransplantation: summary of an NIDDK workshop. Ann Surg. (2015) 261:21–9. 10.1097/SLA.0000000000001059 25599324 PMC4567980

[2] Hwang R Liou P Kato T. *Ex vivo* liver resection and autotransplantation: an emerging option in selected indications. J Hepatol. (2018) 69:1002–3. 10.1016/j.jhep.2018.09.005 30243765

[3] Sudan D. The current state of intestine transplantation: indications, techniques, outcomes and challenges. Am J Transplant. (2014) 14:1976–84. 10.1111/ajt.12812 25307033

[4] Gholam PM Iyer R Johnson MS. Multidisciplinary management of patients with unresectable hepatocellular carcinoma: a critical appraisal of current evidence. Cancers. (2019) 11:873. 10.3390/cancers11060873 31234476 PMC6627394

[5] Kato T Hwang R Liou P Weiner J Griesemer A Samstein B . *Ex vivo* resection and autotransplantation for conventionally unresectable tumors - an 11-year single center experience. Ann Surg. (2020) 272:766–72. 10.1097/SLA.0000000000004270 32833756

[6] Fernández J Alconchel F Gómez B Martínez J Ramírez P. Unresectable GIST liver metastases and liver transplantation: a review and theoretical basis for a new indication. Inte J Surg. (2021) 94:106126. 10.1016/j.ijsu.2021.106126 34592432

[7] Abu-Elmagd K. The concept of gut rehabilitation and the future of visceral transplantation. Nat Rev Gastroenterol Hepatol. (2015) 12:108–20. 10.1038/nrgastro.2014.216 25601664

[8] Liou P Kato T. *Ex vivo* resection and autotransplantation for pancreatic neoplasms. Surg Clin North Am. (2018) 98:189–200. 10.1016/j.suc.2017.09.012 29191274

[9] Yang X Lu L Zhu WW Tao YF Shen CH Chen JH . *Ex vivo* liver resection and auto-transplantation as an alternative for the treatment of liver malignancies: progress and challenges. Hepatobiliary Pancreat Dis Int. (2024) 23:117–22. 10.1016/j.hbpd.2023.10.007 38619051

[10] Wu G. Intestinal autotransplantation. Gastroenterol Rep. (2017) 5:258–65. 10.1093/gastro/gox027 29230296 PMC5691802

[11] Aji T Dong JH Shao YM Zhao JM Li T Tuxun T . *Ex vivo* liver resection and autotransplantation as alternative to allotransplantation for end-stage hepatic alveolar echinococcosis. J Hepatol. (2018) 69:1037–46. 10.1016/j.jhep.2018.07.006 30031886

[12] George A Rammohan A Reddy SM Rela M. *Ex situ* liver resection and autotransplantation for advanced cholangiocarcinoma. BMJ Case Rep. (2019) 12:e230808. 10.1136/bcr-2019-230808 31431431 PMC6706666

[13] Xia P Wang XQ Tian QS Shang-Guan CL Zhu HH. Case report: semi-*ex vivo* hepatectomy combined with autologous liver transplantation for alveolar echinococcosis in children. Am J Trop Med Hyg. (2023) 109:640–4. 10.4269/ajtmh.23-0276 37549899 PMC10484275

[14] Shen S Kong J Qiu Y Zhang S Qin Y Wang W. *Ex vivo* liver resection and autotransplantation versus allotransplantation for end-stage hepatic alveolar echinococcosis. Int J Infect Dis. (2019) 79:87–93. 10.1016/j.ijid.2018.11.016 30496849

[15] Torzilli G. Parenchyma-sparing vessel-guided major hepatectomy: nonsense or new paradigm in liver surgery? Br J Surg. (2021) 108:109–11. 10.1093/bjs/znaa112 33711137

[16] Radulova-Mauersberger O Weitz J Riediger C. Vascular surgery in liver resection. Langenbeck Arch Surg. (2021) 406:2217–48. 10.1007/s00423-021-02310-w 34519878 PMC8578135

[17] Shen S Qiu Y Yang X Wang W. Remnant liver-to-standard liver volume ratio below 40% is safe in *ex vivo* liver resection and autotransplantation. J Gastrointest Surg. (2019) 23:1964–72. 10.1007/s11605-018-4022-4 30374819

[18] Liu W Li G Jin Y Feng Y Gao Z Liu X . Autologous liver transplantation for unresectable hepatobiliary malignancies in enhanced recovery after surgery model. Open Med. (2024) 19:20240926. 10.1515/med-2024-0926 38584830 PMC10998668

[19] Yang X Qiu Y Huang B Wang W Shen S Feng X . Novel techniques and preliminary results of *ex vivo* liver resection and autotransplantation for end-stage hepatic alveolar echinococcosis: a study of 31 cases. Am J Transplant. (2018) 18:1668–79. 10.1111/ajt.14621 29232038 PMC6055796

[20] Hu CL Han X Gao ZZ Zhou B Tang JL Pei XR . Systematic sequential therapy for *ex vivo* liver resection and autotransplantation: a case report and review of literature. World J Gastrointest Surg. (2023) 15:2663–73. 10.4240/wjgs.v15.i11.2663 38111758 PMC10725551

[21] Tai DS Shen N Szot GL Posselt A Feduska NJ Habashy A . Autologous islet transplantation with remote islet isolation after pancreas resection for chronic pancreatitis. JAMA Surg. (2015) 150:118–24. 10.1001/jamasurg.2014.932 25494212

[22] Le Cosquer G Maulat C Bournet B Cordelier P Buscail E Buscail L. Pancreatic cancer in chronic pancreatitis: pathogenesis and diagnostic approach. Cancers. (2023) 15:761. 10.3390/cancers15030761 36765725 PMC9913572

[23] Tariq M Jajja MR Maxwell DW Galindo RJ Sweeney JF Sarmiento JM. Diabetes development after distal pancreatectomy: results of a 10 year series. HPB. (2020) 22:1034–41. 10.1016/j.hpb.2019.10.2440 31718897

[24] Arce KM Lin YK Stevens T Walsh RM Hatipoglu BA. Total pancreatectomy and islet cell autotransplantation: definitive treatment for chronic pancreatitis. Cleve Clin J Med. (2016) 83:435–42. 10.3949/ccjm.83a.15056 27281245

[25] Rickels MR Robertson RP. Pancreatic islet transplantation in humans: recent progress and future directions. Endocr Rev. (2019) 40:631–68. 10.1210/er.2018-00154 30541144 PMC6424003

[26] Cruz RJ Jr McGurgan J Butera L Poloyac K Roberts M Stein W . Gastrointestinal tract reconstruction in adults with ultra-short bowel syndrome: surgical and nutritional outcomes. Surgery. (2020) 168:297–304. 10.1016/j.surg.2019.12.001 32139142

[27] Wu G Zhao L Jiang W Liu C Zhou X Zhang W . Intestinal autotransplantation for locally advanced or locally recurrent colon cancer invading SMA. Ann Surg. (2025) 281:462–8. 10.1097/SLA.0000000000006178 38088199 PMC11809701

[28] Shanbhogue LK Molenaar JC. Short bowel syndrome: metabolic and surgical management. Br J Surg. (1994) 81:486–99. 10.1002/bjs.1800810404 8205419

[29] Goulet O Ruemmele F. Causes and management of intestinal failure in children. Gastroenterology. (2006) 130(2 Suppl 1):S16–28. 10.1053/j.gastro.2005.12.002 16473066

[30] Matsumoto CS Subramanian S Fishbein TM. Adult intestinal transplantation. Gastroenterol Clin North Am. (2018) 47:341–54. 10.1016/j.gtc.2018.01.011 29735028 PMC6433128

[31] Pironi L Staun M Van Gossum A. Intestinal transplantation. N Engl J Med. (2009) 361:2388–9. 10.1056/NEJMc0909351 20007566

[32] Baimas-George M Thompson KJ Watson MD Iannitti DA Martinie JB Baker EH . The technical aspects of *ex vivo* hepatectomy with autotransplantation: a systematic review and meta-analysis. Langenbeck Arch Surg. (2021) 406:2177–200. 10.1007/s00423-021-02093-0 33591451

[33] Zawistowski M Nowaczyk J Jakubczyk M Domagała P. Outcomes of *ex vivo* liver resection and autotransplantation: a systematic review and meta-analysis. Surgery. (2020) 168:631–42. 10.1016/j.surg.2020.05.036 32727659

[34] Kumar R Chung WY Dennison AR Garcea G. Current principles and practice in autologous intraportal islet transplantation: a meta-analysis of the technical considerations. Clin Transplant. (2016) 30:344–56. 10.1111/ctr.12695 26782650

[35] Lu Q Aini A Tang R Dong J. From liver surgery to liver transplant surgery: new developments in autotransplantation. Curr Opin Organ Transplant. (2022) 27:337–45. 10.1097/MOT.0000000000000999 36354260

[36] Ling LX Ouyang Y Hu Y. Research trends on nanomaterials in gastric cancer: a bibliometric analysis from 2004 to 2023. J Nanobiotechnology. (2023) 21:248. 10.1186/s12951-023-02033-8 37533041 PMC10394877

[37] Zhang T Yin X Yang X Man J He Q Wu Q . Research trends on the relationship between microbiota and gastric cancer: a bibliometric analysis from 2000 to 2019. J Cancer. (2020) 11:4823–31. 10.7150/jca.44126 32626529 PMC7330707

[38] Pei Z Chen S Ding L Liu J Cui X Li F . Current perspectives and trend of nanomedicine in cancer: a review and bibliometric analysis. J Control Release. (2022) 352:211–41. 10.1016/j.jconrel.2022.10.023 36270513

[39] Yuan J Chen X Hou L Wang H Zhou Y Pang M . Single-center experience of *ex vivo* liver resection and autotransplantation for complex hepatic alveolar echinoccosis. Front Surg. (2023) 10:1089788. 10.3389/fsurg.2023.1089788 36874451 PMC9975350

[40] Yang C Yang HJ Deng SP Zhang Y. Current status of *ex-vivo* liver resection and autologous liver transplantation for end-stage hepatic alveolar echinococcosis. Ann Palliat Med. (2020) 9:2271–8. 10.21037/apm-20-184 32576011

[41] Bellin MD Freeman ML Gelrud A Slivka A Clavel A Humar A . Total pancreatectomy and islet autotransplantation in chronic pancreatitis: recommendations from PancreasFest. Pancreatology. (2014) 14:27–35. 10.1016/j.pan.2013.10.009 24555976 PMC4058640

[42] Chinnakotla S Radosevich DM Dunn TB Bellin MD Freeman ML Schwarzenberg SJ . Long-term outcomes of total pancreatectomy and islet auto transplantation for hereditary/genetic pancreatitis. J Am Coll Surg. (2014) 218:530–43. 10.1016/j.jamcollsurg.2013.12.037 24655839 PMC4090308

[43] Lv T Xu G Xu X Wu G Wan CF Song JL . A novel remnant liver-first strategy for liver autotransplantation in patients with end-stage hepatic alveolar echinococcosis: a retrospective case series. Int J Surg. (2023) 109:3262–72. 10.1097/JS9.0000000000000604 37994730 PMC10651293

[44] Rostami A Lundström-Stadelmann B Frey CF Beldi G Lachenmayer A Chang BCH . Human alveolar echinococcosis-a neglected zoonotic disease requiring urgent attention. Int J Mol Sci. (2025) 26:2784. 10.3390/ijms26062784 40141427 PMC11943292

[45] Aini A Lu Q Wen H Wang WT Aji T Chen ZY . Particular Chinese contributions to extracorporeal liver surgery. Hepatobiliary Pancreat Dis. (2025) 24:57–66. 10.1016/j.hbpd.2024.12.005 39753427

[46] Abu-El-Haija M Anazawa T Beilman GJ Besselink MG Del Chiaro M Demir IE . The role of total pancreatectomy with islet autotransplantation in the treatment of chronic pancreatitis: a report from the International Consensus Guidelines in chronic pancreatitis. Pancreatology. (2020) 20:762–71. 10.1016/j.pan.2020.04.005 32327370

[47] Coluzzi M Takita M Saracino G Rub Hakim Mohammed A Darden CM Testa G . Improved quality of life among chronic pancreatitis patients undergoing total pancreatectomy with islet autotransplantation-single center experience with large cohort of patients. Transplant Int. (2023) 36:11409. 10.3389/ti.2023.11409 37727384 PMC10505652

[48] Chen ME Desai CS. Current practices in islet cell autotransplantation. Expert Rev Endocrinol Metab. (2023) 18:419–25. 10.1080/17446651.2023.2256407 37680038

[49] Qian NS Liao YH Cai SW Raut V Dong JH. Comprehensive application of modern technologies in precise liver resection. Hepatobiliary Pancreat Dis. (2013) 12:244–50. 10.1016/S1499-3872(13)60040-5 23742768

