# Supplementary material for: Advancements and trends in digestive system autotransplantation: a bibliometric and visualization analysis
Source: Front Med (Lausanne). 2025 Jul 17;12:1537446. doi: 10.3389/fmed.2025.1537446 (PMC12310704; doi:10.3389/fmed.2025.1537446)
Supplement: Supplementary file 1 [file Table_1.docx]

Table S1: Search Strategy for WoSCC Database

| Component | Details |
| --- | --- |
| Database Searched | Web of Science Core Collection (WoSCC) |
| Date of Search | 22-May-24 |
| Time Span | 2004 to 2024 |
| Search Strategy | Concept 1: Autotransplantation Terms (Topic Search - TS) |
|  | TS=(Autotransplantation) OR TS=(&quot;Autologous Transplantation&quot;) OR TS=(&quot;Autologous Transplantations&quot;) OR TS=(&quot;Transplantations, Autologous&quot;) OR TS=(Autografting) OR TS=(Autograftings) OR TS=(Autotransplantation) OR TS=(Autotransplantations) |
|  | AND |
|  | Concept 2: Digestive Organ Terms OR Specific Organ Autotransplantation Terms (Topic Search - TS) |
|  | ( (TS=(Liver) OR TS=(Livers) OR TS=(Pancreas) OR TS=(pancreatic) OR TS=(&quot;Intestine, Small&quot;) OR TS=(&quot;Intestines, Small&quot;) OR TS=(&quot;Small Intestines&quot;) OR TS=(&quot;Small Intestine&quot;) OR TS=(intestine)) |
|  | OR |
|  | (TS=(&quot;autologous liver transplant&quot;) OR TS=(&quot;autologous liver transplantation&quot;) OR TS=(&quot;Autologous Pancreas Transplantation&quot;) OR TS=(&quot;Autologous Pancreas Transplant&quot;) OR TS=(&quot;autologous small intestine transplant&quot;) OR TS=(&quot;Autologous small bowel transplantation&quot;) OR TS=(&quot;intestinal autotransplantation&quot;) OR TS=(&quot;Pancreas autotransplantation&quot;) OR TS=(&quot;intestinal autotransplant&quot;) OR TS=(&quot;Pancreas autotransplant&quot;) OR TS=(&quot;Liver autotransplantation&quot;) OR TS=(&quot;liver autotransplant&quot;)) ) |
| Full Query String (for reference) | ((((((((TS=(Autotransplantation)) OR TS=(&quot;Autologous Transplantation&quot;)) OR TS=(&quot;Autologous Transplantations&quot;)) OR TS=(&quot;Transplantations, Autologous&quot;)) OR TS=(Autografting)) OR TS=(Autograftings)) OR TS=(Autotransplantation)) OR TS=(Autotransplantations)) AND ((((((((((TS=(Liver)) OR TS=(Livers)) OR TS=(Pancreas)) OR TS=(pancreatic)) OR TS=(&quot;Intestine, Small&quot;)) OR TS=(&quot;Intestines, Small&quot;)) OR TS=(&quot;Small Intestines&quot;)) OR TS=(&quot;Small Intestine&quot;))) OR TS=(intestine)) OR (((((((((((TS=(&quot;autologous liver transplant&quot;)) OR TS=(&quot;autologous liver transplantation&quot;)) OR TS=(&quot;Autologous Pancreas Transplantation&quot;)) OR TS=(&quot;Autologous Pancreas Transplant&quot;)) OR TS=(&quot;autologous small intestine transplant&quot;)) OR TS=(&quot;Autologous small bowel transplantation&quot;)) OR TS=(&quot;intestinal autotransplantation&quot;)) OR TS=(&quot;Pancreas autotransplantation&quot;)) OR TS=(&quot;intestinal autotransplant&quot;)) OR TS=(&quot;Pancreas autotransplant&quot;)) OR TS=(&quot;Liver autotransplantation&quot;)) OR TS=(&quot;liver autotransplant&quot;))) |
