# Supplementary material for: Advancements and trends in digestive system autotransplantation: a bibliometric and visualization analysis
Source: Front Med (Lausanne). 2025 Jul 17;12:1537446. doi: 10.3389/fmed.2025.1537446 (PMC12310704; doi:10.3389/fmed.2025.1537446)
Supplement: Supplementary file 2 [file Table_2.docx]

Table S2: Publication output of the top 10 countries/regions in the study of autotransplantation for the digestive system.

| Rank | Country/region | Article counts | centrality | Percentage (%) | Citation | Citation per publication |
| --- | --- | --- | --- | --- | --- | --- |
| 1 | USA | 300 | 0.54 | 40.11% | 7323 | 24.41 |
| 2 | CHINA | 172 | 0.01 | 22.99% | 2561 | 14.89 |
| 3 | ITALY | 57 | 0.09 | 7.62% | 2093 | 36.72 |
| 4 | JAPAN | 48 | 0.08 | 6.42% | 1668 | 34.75 |
| 5 | ENGLAND | 29 | 0.15 | 3.88% | 1056 | 36.41 |
| 6 | BRAZIL | 24 | 0 | 3.21% | 204 | 8.50 |
| 7 | GERMANY | 23 | 0.09 | 3.07% | 728 | 31.65 |
| 8 | CANADA | 21 | 0.07 | 2.81% | 408 | 19.43 |
| 9 | FRANCE | 19 | 0.03 | 2.54% | 1341 | 70.58 |
| 10 | SOUTH KOREA | 19 | 0 | 2.54% | 351 | 18.47 |
