# Supplementary material for: Advancements and trends in digestive system autotransplantation: a bibliometric and visualization analysis
Source: Front Med (Lausanne). 2025 Jul 17;12:1537446. doi: 10.3389/fmed.2025.1537446 (PMC12310704; doi:10.3389/fmed.2025.1537446)
Supplement: Supplementary file 3 [file Table_3.docx]

Table S3: Publication output of the top 10 institutions in the study of autotransplantation for the digestive system.

| Rank | Institution | Country | Number of studies | Total citations | Average citation |
| --- | --- | --- | --- | --- | --- |
| 1 | University of Minnesota System | USA | 71 | 2258 | 31.80 |
| 2 | University of Minnesota Twin Cities | USA | 70 | 2101 | 30.01 |
| 3 | University System of Ohio | USA | 31 | 350 | 11.29 |
| 4 | Cincinnati Children's Hospital Medical Center | USA | 23 | 227 | 9.87 |
| 5 | Pennsylvania Commonwealth System of Higher Education (PCSHE) | USA | 23 | 836 | 36.35 |
| 6 | University of Pittsburgh | USA | 22 | 765 | 34.77 |
| 7 | Xinjiang Medical University | China | 22 | 666 | 30.27 |
| 8 | Harvard University | USA | 21 | 305 | 14.52 |
| 9 | Baylor University Medical Center | USA | 21 | 235 | 11.19 |
| 10 | Sichuan University | China | 21 | 172 | 8.19 |
