# Supplementary material for: Advancements and trends in digestive system autotransplantation: a bibliometric and visualization analysis
Source: Front Med (Lausanne). 2025 Jul 17;12:1537446. doi: 10.3389/fmed.2025.1537446 (PMC12310704; doi:10.3389/fmed.2025.1537446)
Supplement: Supplementary file 4 [file Table_4.docx]

Table S4: Data on publication volume within the top 10 journals dedicated to research on autotransplantation for the digestive system.

| Rank | Journal | Article counts | Percentage（748） | IF | Quartile in category |
| --- | --- | --- | --- | --- | --- |
| 1 | american journal of transplantation | 30 | 4.01% | 8.8 | Q1 |
| 2 | transplantation proceedings | 30 | 4.01% | 0.9 | Q4 |
| 3 | transplantation | 19 | 2.54% | 6.2 | Q1 |
| 4 | pancreatology | 18 | 2.41% | 3.6 | Q2 |
| 5 | world journal of gastroenterology | 17 | 2.27% | 4.3 | Q2 |
| 6 | cell transplantation | 16 | 2.14% | 3.3 | Q2 |
| 7 | journal of gastrointestinal surgery | 16 | 2.14% | 3.2 | Q3 |
| 8 | pancreas | 16 | 2.14% | 2.9 | Q3 |
| 9 | surgery | 14 | 1.87% | 3.8 | Q1 |
| 10 | annals of surgery | 13 | 1.74% | 10.1 | Q1 |
