# Supplementary material for: Advancements and trends in digestive system autotransplantation: a bibliometric and visualization analysis
Source: Front Med (Lausanne). 2025 Jul 17;12:1537446. doi: 10.3389/fmed.2025.1537446 (PMC12310704; doi:10.3389/fmed.2025.1537446)
Supplement: Supplementary file 5 [file Table_5.docx]

Table S5: Co-citation table of the top 10 journals in the study of autotransplantation for the digestive system.

| Rank | Cited Journal | Co-Citation | IF（2022） | Quartile in category |
| --- | --- | --- | --- | --- |
| 1 | ANN SURG | 362 | 10.1 | Q1 |
| 2 | TRANSPLANTATION | 338 | 6.2 | Q1 |
| 3 | SURGERY | 300 | 3.8 | Q1 |
| 4 | AM J TRANSPLANT | 275 | 8.8 | Q1 |
| 5 | J AM COLL SURGEONS | 272 | 5.2 | Q1 |
| 6 | NEW ENGL J MED | 258 | 158.5 | Q1 |
| 7 | PANCREAS | 250 | 2.9 | Q3 |
| 8 | J GASTROINTEST SURG | 223 | 3.2 | Q3 |
| 9 | DIABETES | 218 | 7.7 | Q1 |
| 10 | GASTROENTEROLOGY | 198 | 29.4 | Q1 |
