# Supplementary material for: Advancements and trends in digestive system autotransplantation: a bibliometric and visualization analysis
Source: Front Med (Lausanne). 2025 Jul 17;12:1537446. doi: 10.3389/fmed.2025.1537446 (PMC12310704; doi:10.3389/fmed.2025.1537446)
Supplement: Supplementary file 6 [file Table_6.docx]

Table S6: Top 10 authors and co-cited authors related to the study of autotransplantation for the digestive system.

| Rank | Author | Count | Rank | Co-cited author | Citation |
| --- | --- | --- | --- | --- | --- |
| 1 | bellin, melena d. | 43 | 1 | BELLIN MD | 171 |
| 2 | freeman, martin l. | 23 | 2 | SUTHERLAND DER | 163 |
| 3 | chinnakotla, srinath | 22 | 3 | CHINNAKOTLA S | 100 |
| 4 | naziruddin, bashoo | 22 | 4 | RICORDI C | 88 |
| 5 | pruett, timothy l. | 22 | 5 | SHAPIRO AMJ | 80 |
| 6 | beilman, gregory j. | 19 | 6 | AHMAD SA | 60 |
| 7 | dunn, ty b. | 19 | 7 | WILSON GC | 55 |
| 8 | schwarzenberg, sarah j. | 18 | 8 | ROBERTSON RP | 50 |
| 9 | sutherland, david e. r. | 18 | 9 | PICHLMAYR R | 48 |
| 10 | chi, xinjin | 17 | 10 | BALZANO G | 43 |
