# Supplementary material for: Advancements and trends in digestive system autotransplantation: a bibliometric and visualization analysis
Source: Front Med (Lausanne). 2025 Jul 17;12:1537446. doi: 10.3389/fmed.2025.1537446 (PMC12310704; doi:10.3389/fmed.2025.1537446)
Supplement: Supplementary file 7 [file Table_7.docx]

Table S7: Top 10 co-cited references related to autotransplantation for the digestive system.

| Rank | Title | Journal | author(s) | Total citations |
| --- | --- | --- | --- | --- |
| 1 | Total Pancreatectomy and Islet Autotransplantation for Chronic Pancreatitis | *JOURNAL OF THE AMERICAN COLLEGE OF SURGEONS* | Sutherland DER | 65 |
| 2 | Total Pancreatectomy and Islet Autotransplantation in Children for Chronic Pancreatitis Indication, Surgical Techniques, Postoperative Management, and Long-Term Outcomes | *ANNALS OF SURGERY* | Chinnakotla S | 36 |
| 3 | Ex vivo liver resection and autotransplantation as alternative to allotransplantation for end-stage hepatic alveolar echinococcosis | *JOURNAL OF HEPATOLOGY* | Aji T | 31 |
| 4 | Factors Predicting Outcomes After a Total Pancreatectomy and Islet Autotransplantation Lessons Learned From Over 500 Cases | *ANNALS OF SURGERY* | Chinnakotla S | 30 |
| 5 | Total pancreatectomy and islet autotransplantation in chronic pancreatitis: Recommendations from PancreasFest | *PANCREATOLOGY* | Bellin MD | 29 |
| 6 | Total Pancreatectomy With Islet Autotransplantation Resolves Pain in Young Children With Severe Chronic Pancreatitis | *JOURNAL OF PEDIATRIC GASTROENTEROLOGY AND NUTRITION* | Bellin MD | 26 |
| 7 | Ex Vivo Liver Resection and Autotransplantation for End-Stage Alveolar Echinococcosis: A Case Series | *AMERICAN JOURNAL OF TRANSPLANTATION* | Wen H | 25 |
| 8 | Systematic review of total pancreatectomy and islet autotransplantation for chronic pancreatitis | *BRITISH JOURNAL OF SURGERY* | Bramis K | 24 |
| 9 | How Durable Is Total Pancreatectomy and Intraportal Islet Cell Transplantation for Treatment of Chronic Pancreatitis? | *JOURNAL OF THE AMERICAN COLLEGE OF SURGEONS* | Bellin MD | 24 |
| 10 | Novel techniques and preliminary results of ex vivo liver resection and autotransplantation for end-stage hepatic alveolar echinococcosis: A study of 31 cases | *AMERICAN JOURNAL OF TRANSPLANTATION* | Yang XW | 23 |
