# Supplementary material for: Advancements and trends in digestive system autotransplantation: a bibliometric and visualization analysis
Source: Front Med (Lausanne). 2025 Jul 17;12:1537446. doi: 10.3389/fmed.2025.1537446 (PMC12310704; doi:10.3389/fmed.2025.1537446)
Supplement: Supplementary file 8 [file Table_8.docx]

Table S8: Top 20 keywords associated with autotransplantation for the digestive system.

| Rank | Keyword | Counts | Rank | Keyword | Counts |
| --- | --- | --- | --- | --- | --- |
| 1 | total pancreatectomy | 117 | 11 | insulin | 45 |
| 2 | islet autotransplantation | 100 | 12 | pancreatectomy | 41 |
| 3 | chronic pancreatitis | 95 | 13 | quality-of-life | 41 |
| 4 | management | 71 | 14 | diabetes | 38 |
| 5 | outcm | 63 | 15 | diagnosis | 34 |
| 6 | islet transplantation | 61 | 16 | in-vitro | 34 |
| 7 | chronic-pancreatitis | 53 | 17 | pain | 33 |
| 8 | surgery | 53 | 18 | risk-factors | 32 |
| 9 | resection | 48 | 19 | differentiation | 31 |
| 10 | autologous transplantation | 47 | 20 | expression | 31 |
